# Academic Stress, Family Relationships, and Social Media Use Disorder Among Turkish Middle School Students: Explanatory Sequential Mixed Methods Study

**DOI:** 10.2196/97014

**Published:** 2026-09-22

**Authors:** Ibrahim Arpaci, Vedat Bakır, Ismail Kusci, Mustafa Baloglu

**Affiliations:** 1 Computer Engineering Bursa Uludağ Üni̇versi̇tesi̇ Bursa, Bursa Province Turkey; 2 Korea University Seoul Democratic People's Republic of Korea; 3 Kütahya Dumlupınar University Kütahya, Kütahya Turkey; 4 Tokat Gaziosmanpaşa Üniversitesi Tokat, Tokat Province Turkey; 5 Special and Gifted Education United Arab Emirates University Al Ain, Abu Dhabi United Arab Emirates

**Keywords:** academic stress, adolescents, family relationships, sequential mixed method, social media use disorder

## Abstract

**Background:**

Social media use disorder (SMUD) has become an important concern in adolescent digital health. Although prior research has linked problematic social media use to psychological and family-related factors, less is known about how academic stress and different patterns of family relationships are associated with SMUD among middle school students.

**Objective:**

This study examined the associations among academic stress, restrictive and supportive family relationships, and SMUD among Turkish middle school students. It also explored how students described the role of family relationships and academic pressure in their social media use.

**Methods:**

An explanatory sequential mixed methods design was used. The quantitative phase included 311 middle school students aged 11-14 years. Participants completed paper-and-pencil measures of academic stress, family relationships, and SMUD. Structural equation modeling was used to test the hypothesized indirect pathways, and indirect effects were examined with 5000 bootstrap samples. The qualitative phase included a focus group with 10 students selected from the quantitative sample. Thematic analysis was used to contextualize and refine the quantitative findings.

**Results:**

Academic stress was positively associated with restrictive family relationships (*β*=.679; *P*<.001) and SMUD (*β*=.234; *P*=.03). Restrictive family relationships were also positively associated with SMUD (*β*=.549; *P*=.006). The indirect pathway from academic stress to SMUD through restrictive family relationships was statistically significant (*β*=.373, 95% CI 0.140-0.617; *P*=.004). In contrast, the indirect pathway through supportive family relationships was not significant (*β*=–.056, 95% CI –0.279 to 0.159; *P*=.59). The model explained 42.6% of the variance in SMUD. Qualitative findings showed that students described social media as a source of entertainment, connection, academic support, and stress relief. Some students linked parental restriction and criticism to their social media experiences, while others reported reducing social media use during examination periods to focus on schoolwork.

**Conclusions:**

The findings suggest that academic stress and restrictive family relationships are associated with SMUD among middle school students. The qualitative results add nuance by showing that academic pressure may be linked both to stress-related social media use and, for some students, short-term self-regulation during examinations. These findings support the need for adolescent digital health approaches that consider school pressure, family communication, and balanced parental guidance rather than focusing only on individual screen use.

## Introduction

### Overview

The rapid development and proliferation of digital technologies have rendered social media usage an indispensable part of daily life, particularly among young people [1]. Current statistics indicate that 95% of adolescents aged 13-17 years worldwide actively use at least one social media platform [2]. This widespread adoption has intensified research interest in the multifaceted impacts of social media on young people [3]. In recent years, increasing attention has focused not only on adolescents’ general social media use but also on problematic patterns such as social media addiction and social media use disorder (SMUD), particularly in relation to psychological well-being, academic functioning, and family relationships [4]. In the field of psychology, prior research has established the correlation between social media addiction and both depression and anxiety [5], while in educational sciences, previous research has investigated the influence of social media use on academic performance [6].

In the international context, the effects of social media use vary across cultures. Collectivist cultures, particularly in Asia and Africa, use social media to strengthen social cohesion and family relationships [7], whereas in individualistic cultures, primarily in North America and Europe, social media serves more as a tool for individual expression and personal branding [8]. These distinctions shape individuals’ online behaviors and social interactions, implying that the influence of social media use may vary across cultural contexts.

Bronfenbrenner’s [9] ecological systems theory (EST) provides a comprehensive framework for understanding these complex relationships. The theory conceptualizes individual development within nested environmental systems, including the microsystem (eg, family and school), mesosystem (eg, family-school interaction), exosystem (eg, indirect environmental influences), and macrosystem (eg, cultural context). Applied to adolescent social media behavior, EST helps situate digital engagement within the broader interplay of family, school, and sociocultural contexts. Despite the relevance of this framework, important gaps remain. Existing research has more often focused on broad problematic internet use or generalized adolescent outcomes than on SMUD specifically among middle school students. In particular, limited attention has been paid to how academic stress and differentiated family relationship patterns may interact within an ecological framework during this critical developmental stage.

Middle school is a critical stage when an individual’s psychological, social, and academic development progresses rapidly. This period encompasses identity formation, social skill development, and the navigation of a crucial phase in students’ educational journey. The middle school years play a fundamental role in children’s individual and social development. The developmental experiences during this period can significantly contribute to children’s preparation for future challenges and their subsequent psychological well-being [10]. Research indicates that social and academic transitions experienced during middle school can influence individuals’ long-term development [11]. During this period, while adolescents become more autonomous in their peer relationships, they also experience significant changes in family dynamics. Educational expectations and social interactions can directly impact children’s psychological and social development.

Relatively limited research has integrated middle school students’ SMUD, family relationships, and academic stress within a unified EST framework. To address this gap, the present research uses a mixed methods approach to examine these complex relationships in depth. This study examines the relationships between SMUD, family relationships, and academic stress from an EST perspective among middle school students. The mixed methods approach enables a comprehensive examination of the subject’s multidimensional nature by combining the generalizability of quantitative data with the depth of qualitative data. While quantitative analyses reveal general patterns in relationships between variables, qualitative data will help illuminate the underlying mechanisms and contextual factors that shape these relationships.

This study extends prior EST-based research by specifically examining SMUD, academic stress, and family relational dynamics during middle school. In contrast, this study integrates academic stress, restrictive and supportive family relationship patterns, and SMUD into a unified explanatory model.

In conclusion, this study can potentially guide future research and practice by emphasizing the importance of multidimensional approaches necessary to support healthy youth development in the digital age. Findings from the EST perspective may contribute to a broader understanding of the complex factors shaping young people’s social media use and to the development of effective intervention strategies.

From a digital health perspective, this study contributes to understanding SMUD as an adolescent health-related digital behavior embedded in school and family contexts. Although the study does not evaluate a digital intervention or analyze platform-generated behavioral traces, its findings may inform the design of family-mediated digital well-being interventions, school-based prevention programs, and platform-level strategies that support healthier social media engagement among adolescents. By identifying academic stress and restrictive family relationships as correlates of SMUD, the study highlights contextual targets that may be considered in future digital health prevention and monitoring approaches.

### Theoretical Background

SMUD, a rapidly emerging phenomenon of the digital age, has become an increasingly significant concern due to its potential adverse effects on individuals’ psychological health and social relationships. EST, a comprehensive approach examining individual development within environmental interactions, provides a robust theoretical framework for understanding this complex phenomenon [12]. The EST conceptualizes environmental factors that affect individual development as nested systems. These systems are organized in layers, starting with the microsystem (close environments, such as school and family), followed by the mesosystem (interactions among microsystems), the exosystem (larger social structures that indirectly influence individuals), and the macrosystem (cultural values and policies). This theoretical perspective is well-suited to understanding the multidimensional nature of SMUD, as it enables a comprehensive examination of the environmental factors influencing individual behavior.

While SMUD has not yet received official recognition in *Diagnostic and Statistical Manual of Mental Disorders, Fifth Edition*, it represents an increasingly studied phenomenon among researchers. Kuss and Griffiths [13] define this disorder as excessive and uncontrolled social media use that adversely affects individuals’ daily lives, relationships, and overall well-being. This definition emphasizes how social media use can influence interactions within an individual’s microsystem (eg, family relationships) and mesosystem (eg, academic performance). Recent research has revealed robust associations between SMUD and various psychological and social factors. For instance, Marino et al [14] conducted a meta-analysis that demonstrated significant positive correlations between social media addiction and individual factors such as low self-esteem, loneliness, and depression. In the same vein, individual differences in attachment have been linked to problematic technology use, with mindfulness operating as a mediating mechanism. These findings illuminate how an individual’s internal psychological state can influence social media use behaviors, which can be examined at the microsystem level of EST.

The quality of adolescents’ perceived family relational dynamics and parent-child communication has emerged as a crucial factor influencing social media use at the microsystem level. A prior study demonstrated the significant impact of family functioning and parent-child communication on problematic social media use [15]. These findings highlight how family dynamics can influence individual online behaviors, underscoring the importance of the family microsystem in EST. Academic stress, conceptualized in this study as a school-related microsystem factor, is strongly associated with SMUD. Jun and Choi [16] found that academic stress significantly increases problematic smartphone use among university students. Kardefelt-Winther [17] proposed that online activities may serve as stress-coping mechanisms. This perspective suggests that school-related academic stress and family relational dynamics may jointly shape individual social media use behaviors within a broader EST-informed framework.

Within this theoretical framework, examining the effects of family relationships and academic stress on SMUD among youth helps contextualize these associations within an EST-informed framework, particularly at the microsystem level, while offering cautious theoretical implications for broader system interactions. This understanding may facilitate the development of more effective, holistic intervention strategies to prevent and treat SMUD. The multilayered structure of EST enables the examination of complex relationships among variables. In this context, understanding how family relationships and school-related academic stress may jointly shape individual social media use behaviors is essential. A prior study examined the impact of ecological factors on adolescents’ internet addiction and identified family functioning as a significant mediator [18]. This finding suggests that family relationships may mediate the association between stress factors and problematic internet use. Family relationships are crucial at the microsystem level of EST. In this study, family relationships specifically refer to adolescents’ perceived restrictive and supportive relational dynamics within family interactions, rather than to broader family functioning, emotional climate, or formal parenting-style constructs. Although this study specifically measures adolescents’ perceived family relationship patterns rather than parenting styles directly, Baumrind’s [19] framework provides a useful conceptual lens for interpreting restrictive and supportive relational dynamics within the family context.

### Hypotheses

#### Restrictive Family Relationships

Restrictive family relationships, as conceptualized in this study, may share characteristics with authoritarian or neglect-oriented relational patterns, particularly regarding control, limited autonomy, and reduced emotional support. Stringent rules and elevated expectations characterize these relationships, yet they exhibit a notable absence of emotional support and warmth. Open communication and autonomy are systematically constrained, with punitive disciplinary approaches predominating. An empirical investigation indicated a significant relation between low family functionality and problematic social media use [15]. Similarly, Shek and Yu’s [20] longitudinal investigation of Hong Kong adolescents demonstrated that perceived negative parent-child relationships significantly increase internet addiction risk. Based on this empirical evidence, we propose the following:

- H1: Restrictive family relationships positively predict SMUD.

#### Supportive Family Relationships

Supportive family relationships in this study conceptually overlap with certain dimensions commonly associated with authoritative parenting, such as emotional support, open communication, and autonomy support, while remaining distinct from formal parenting style classifications. These relationships are distinguished by high levels of emotional support and warmth and by fostering open communication and mutual respect. Age-appropriate autonomy and independence are actively encouraged, accompanied by consistent and equitable disciplinary approaches. Schneider et al’s [21] investigation demonstrated that high-quality parent-child relationships are significantly correlated with reduced problematic internet use among adolescents. Wu et al [22] established that family cohesion and parental support significantly decrease internet addiction risk among adolescents. Furthermore, a study by Gunuc and Dogan [23] on Turkish adolescents found a significant negative correlation between perceived parental support and internet addiction. Based on this cumulative empirical evidence, we hypothesize:

- H2: Supportive family relationships negatively predict SMUD.

#### Academic Stress and SMUD

Academic stress encompasses the intense psychological pressure and anxiety students experience in meeting educational demands. This stress manifests in multiple ways: intensive course loads, examination pressure, heightened expectations for academic achievement, career-related anxiety, time-management challenges, and competitive academic environments. Jun and Choi’s [16] research established that academic stress significantly predicts increased problematic smartphone use among university students [16]. Another study found that academic stress significantly increases the risk of social media addiction among university students [24]. Similarly, Wang et al [25] examined Chinese university students and identified depression as a significant mediator in the relationship between academic stress and problematic social media use [25]. Building on this empirical foundation, we propose the following:

- H3: Academic stress positively predicts SMUD.

These hypotheses aim to examine the systematic influence of dynamics occurring at different levels of EST on individual social media use behaviors. Understanding the effects of family relationship quality (microsystem) and academic stress (school-related microsystem factor) on youth SMUD may facilitate the development of more effective, comprehensive intervention strategies. Additionally, the mediating role of family relationships in the relationship between academic stress and SMUD represents a crucial research focus. Shek and Yu’s [20] longitudinal investigation demonstrated that negative parent-child relationships significantly predict increased internet addiction risk. Similarly, Wang et al [25] identified depression as a key mediator in the relationship between academic stress and problematic social media use. These findings highlight the crucial mediating roles of psychological and familial factors in linking stressors to problematic internet and social media use. Based on this theoretical framework and existing empirical evidence, we propose the following additional hypotheses:

- H4: Restrictive family relationships mediate the relationship between academic stress and SMUD.
- H5: Supportive family relationships mediate the relationship between academic stress and SMUD.

## Methods

### Research Design

This study used an “explanatory sequential mixed methods” design. The initial phase used quantitative data collection methods, using standardized scales, to explore relationships among SMUD, family relationships, and academic stress among middle school students. Quantitative data underwent statistical analysis to identify relationships and patterns between variables [26,27].

The subsequent phase involved collecting qualitative data using a semistructured focus group interview guide to gather in-depth information from participants [28]. Qualitative data underwent thematic analysis to explain, contextualize, and formally triangulate quantitative findings through sequential explanatory integration [29]. Specifically, qualitative themes were examined in relation to significant, nonsignificant, and mediating quantitative pathways to generate integrated meta-inferences. This research design facilitates the identification of general trends through comprehensive quantitative analyses and the examination of underlying mechanisms through qualitative inquiry [30].

### Participants and Procedure

The study used paper-and-pencil scales to ensure standardized data collection. Participants were recruited through a convenience sampling procedure after the required institutional and school permissions had been obtained. Schools were approached through the relevant administrative permission process, and students who met the age and grade-level criteria were invited to participate during the data collection period. Participation was voluntary, and only students with consent from a parent or legal guardian were included. Because the sample was not randomly selected, the findings should be interpreted with caution in relation to broader adolescent populations. The quantitative sample consisted of 311 students aged 11-14 years. The qualitative phase included 10 students who voluntarily participated in a focus group and were purposively selected from the quantitative sample to provide diverse perspectives on social media use, family relationships, and academic stress.

### Data Collection

#### SMUD

SMUD, developed by van Den Eijnden et al [31], was adapted to Turkish by Sariçam and Adam [32]. The internal consistency coefficient is 0.75, with a split-half reliability of 0.64. “Confirmatory factor analysis” (CFA) revealed good model fit indices. The unidimensional scale indicates that higher scores correspond to a greater likelihood of SMUD. Factor loadings ranged from 0.35 to 0.76, highly correlated with the “Young Internet Addiction Test-Short Form” (*r*=0.64). Cronbach α is 0.75, with item-total correlation coefficients ranging from 0.29 to 0.73. This study found Cronbach α to be 0.85. In this study, SMUD was treated as a continuous indicator of symptom severity rather than as a dichotomous disorder/nondisorder classification. Therefore, no cutoff score was applied. This approach was consistent with the study’s aim of examining model-based associations among academic stress, family relationship patterns, and SMUD severity within the structural equation modeling (SEM) framework. No items were removed from the SMUD scale. The established unidimensional structure was retained to preserve comparability with the Turkish adaptation and previous studies using the scale. Although the lower factor loadings reported in the original validation study suggest that some items may represent the latent construct less strongly than others, the reliability coefficient observed in the present sample was acceptable.

#### Family Relationships Scale for Children

The Family Relationships Scale for Children (FRSC), developed by Zorbaz and Owen [33], assesses children’s perceptions of family relational patterns, specifically restrictive and supportive dynamics, rather than broader family functioning or formal parenting style classifications. “Exploratory factor analysis” reduced the scale from 56 to 20 items, revealing a 2-factor structure: “Restrictive Family Relationships” and “Supportive Family Relationships.” CFA yielded adequate model fit indices. Cronbach α coefficients were 0.82 for “Restrictive Family Relationships” and 0.76 for “Supportive Family Relationships.” The scale uses a “3-point Likert-type” response format, analyzing separate scores for both subscales without calculating a total score. This study calculated Cronbach α coefficients as 0.80 for “Restrictive Family Relationships” and 0.85 for “Supportive Family Relationships.”

#### Educational Stress Scale

The Educational Stress Scale was developed by Sun et al [34] and adapted into Turkish by Celik et al [35]. CFA validated the 5-factor structure of 16 items, indicating a good model fit. Cronbach α coefficient was 0.86 for the overall scale and ranged from 0.87 to 0.93 for subscales. The test-retest reliability coefficient was 0.67, with item-total correlations ranging from 0.40 to 0.57. In this study, the Cronbach α coefficient was found to be 0.79.

### Focus Group

The focus group methodology was selected for its effectiveness in generating rich, qualitative data through participant interaction [26]. This approach facilitates an in-depth exploration of perspectives through structured group discussions [27]. Focus groups enable efficient data collection while allowing detailed examination of specific topics through guided discussion [36]. The semistructured interview guide was developed through consultation with 3 experts in guidance, psychological counseling, and child development, ensuring content validity and comprehensive coverage of research objectives.

### Data Analysis

#### Quantitative Data Analysis

The quantitative analysis protocol was conducted in SPSS (version 28; IBM Corp) using a systematic analytical framework. The preliminary analysis phase began by evaluating the data distribution using skewness and kurtosis to assess the normality assumption. Following confirmation of the distributional properties, the analysis examined the relationships among key variables (restrictive family relationships, supportive family relationships, academic stress, and SMUD) using Pearson correlation coefficients.

The mediation analysis was conducted using AMOS (version 28; IBM Corp) software within an SEM framework. The model incorporated academic stress as the independent variable, restrictive and supportive family relationships as mediating variables, and SMUD as the dependent variable. The significance of indirect effects was rigorously tested via a bootstrap analysis with 5000 samples and a 95% CI. Model fit assessment used multiple indices: “Chi-square statistic,” “Comparative Fit Index” (CFI), “Tucker-Lewis Index” (TLI), “root-mean-square error of approximation” (RMSEA), and “standardized root-mean-square residual” (SRMR), ensuring comprehensive evaluation of model adequacy.

#### Qualitative Data Analysis

The qualitative analysis used a comprehensive strategy combining descriptive and thematic approaches. Initial data preparation involved meticulous transcription of participants’ responses regarding their experiences with social media use, family relationships, and academic stress. These transcripts underwent a thorough preliminary examination for content and meaning before the commencement of formal coding procedures.

The process incorporated open coding for direct content analysis and action coding for behavioral and interactional elements. This dual approach facilitated the identification and development of central themes and subthemes, followed by a systematic study of the thematic relationships. Qualitative data were coded by multiple researchers, who compared coding patterns and resolved discrepancies through discussion and consensus to strengthen coding consistency and analytical rigor. Given the explanatory purpose of the qualitative phase and the use of one focus group, the analysis was evaluated in terms of thematic sufficiency rather than broad saturation. The qualitative material was considered sufficient for contextualizing the quantitative findings when no substantially new explanatory patterns emerged during repeated coding discussions.

The integrated analysis revealed comprehensive insights into the interrelationships between social media use patterns, family dynamics, and academic stress within students’ lived experiences. This analytical approach facilitated a profound understanding of how these factors impact student well-being and development. The findings offered nuanced insights into the complex relationships between digital media engagement, family relationships, and academic pressures in contemporary student life.

### Ethical Considerations

This study was approved by the Social and Human Sciences Scientific Research and Publication Ethics Committee of Kütahya Dumlupınar University (meeting number 2024/04; meeting date: April 29, 2024; decision number 26; document date and number: May 7, 2024-214). Consent from a parent or legal guardian was obtained for participants aged <16 years, and student participation was voluntary. Required permissions for data collection were obtained from the relevant Provincial Directorate of National Education.

## Results

### Correlations and Descriptive Statistics

As Table 1 shows, correlation analyses revealed significant associations among all study variables (*P*<.001). Specifically, academic stress demonstrated significant positive correlations with restrictive family relationships (*r*=0.450) and SMUD (*r*=0.346). Conversely, academic stress was significantly and negatively correlated with supportive family relationships (*r*=–0.372). Similarly, SMUD showed a significant positive correlation with restrictive family relationships (*r*=0.499) and a significant negative correlation with supportive family relationships (*r*=–0.444). These correlation coefficients indicate moderate-to-strong relationships among the study variables, suggesting substantial interconnections among academic stress, family relationship dynamics, and social media use patterns.

**Table 1:** Descriptive statistics and Pearson correlations.

Variable			Mean (SD)		Cronbach α		McDonald ω		Restrictive family relationships		Supportive family relationships		Social media use disorder		Academic stress
Restrictive family relationships															
	*r*	1.50 (0.38)		0.798		0.798		1							
	*P* value	—^a^		—		—		—							
Supportive family relationships															
	*r*	2.62 (0.38)		0.847		0.850		–0.679		1					
	*P* value	—		—		—		<.001		—					
Social media use disorder															
	*r*	1.63 (0.38)		0.846		0.846		0.499		–0.444		1			
	*P* value	—		—		—		<.001		<.001		—			
Academic stress															
	*r*	2.96 (0.67)		0.788		0.779		0.450		–0.372		0.346		1	
	*P* value	—		—		—		<.001		<.001		<.001		—	

^a^Not applicable.

### Measurement Model

We evaluated the measurement model’s validity before conducting SEM. Scale reliability assessment used Cronbach α along with McDonald ω coefficients. All scales demonstrated robust reliability coefficients (α and ω >0.70): supportive family relationships (Cronbach α=0.847; McDonald ω=0.850), restrictive family relationships (Cronbach α=0.798; McDonald ω=0.798), academic stress (Cronbach α=0.788; McDonald ω=0.779), and SMUD (Cronbach α=0.846; McDonald ω=0.846).

The measurement model exhibited excellent fit indices (*χ*^2^_513_=795.653; *P*<.001; *χ*^2^_513_=1.551; CFI=0.911; TLI=0.902; RMSEA=0.042, 90% CI 0.036-0.048; SRMR=0.073). All indicator factor loadings were statistically significant (*P*<.001) and exceeded the 0.30 threshold (range 0.339-0.745), confirming the robustness of the measurement model.

### Structural Model and Mediation Analysis

Following measurement model confirmation, analysis of the proposed structural model revealed comparable fit indices (*χ*^2^_513_=795.653; *P*<.001; *χ*^2^_513_=1.551; CFI=0.911; TLI=0.902; RMSEA=0.042, 90% CI 0.036-0.048; SRMR=0.073; Table 2).

Path analysis indicated that academic stress significantly predicted both supportive (*β*=–.610; *P*<.001) and restrictive (*β*=.679; *P*<.001) family relationships. Academic stress showed a significant direct association with SMUD (*β*=.234; *P*=.03). Restrictive family relationships were significantly associated with SMUD (*β*=.549; *P*=.006), whereas supportive family relationships were not (*β*=.091; *P*=.58).

Bootstrap analysis (5000 samples) indicated a significant total indirect effect of academic stress on SMUD (*β*=.317, 95% CI 0.174-0.536; *P*<.001). Examination of specific indirect pathways indicated that academic stress exerted a significant indirect effect on SMUD through restrictive family relationships (*β*=.373, 95% CI 0.140-0.617; *P*=.004). However, the indirect pathway through supportive family relationships did not reach statistical significance (*β*=–.056, 95% CI –0.279 to 0.159; *P*=.59).

The total association between academic stress and SMUD, encompassing both direct and indirect pathways, achieved significance (*β*=.551, 95% CI 0.436-0.658; *P*<.001). The model accounted for 42.6% of the variance in SMUD (*R*^2^=0.426). These findings were consistent with a partial indirect pathway from academic stress to SMUD through restrictive family relationships within the hypothesized cross-sectional model. Specifically, higher academic stress was associated with higher restrictive family relationships, which were in turn associated with greater SMUD vulnerability. Notably, supportive family relationships did not have a significant mediating effect in this relationship.

**Table 2:** Mediation analysis.

Path			*β*		SE		*P* value		95% CI
Direct effects									
	Academic stress → supportive family	–.610		0.053		<.001		–0.711 to –0.500	
	Academic stress → restrictive family	.679		0.056		<.001		0.560 to 0.781	
	Academic stress → SMUD^a^	.234		0.116		.03		0.019 to 0.442	
	Supportive family → SMUD	.091		0.214		.58		–0.279 to 0.559	
	Restrictive family → SMUD	.549		0.248		.006		0.140 to 1.126	
Indirect effects									
	Academic stress → SMUD (total)	.317		0.088		<.001		0.174 to 0.536	
	Via supportive family	–.056		—		.59		–0.279 to 0.159	
	Via restrictive family	.373		—		.004		0.140 to 0.617	
Total effect									
	Academic stress → SMUD	.551		0.058		<.001		0.436 to 0.658	

^a^SMUD: social media use disorder.

### Qualitative Findings

#### Overview

The qualitative findings were organized around 3 themes: social media use in daily life, family responses to social media use, and academic stress and regulation. These themes were used to contextualize the quantitative findings and to clarify how students described the links among academic stress, family relationships, and SMUD.

#### Social Media Usage Patterns

The qualitative interviews with students were structured around key themes, including social media use, family relationships, and academic stress. Social media emerged as both an entertainment and research tool for students. Most participants had integrated social media platforms into their daily routines, with usage patterns varying significantly among respondents. Several participants reported frequent engagement with social media. For instance, participant 1 described integrating social media into their daily routine: “I generally spend most of my time on social media.” Some students, such as participant 9, reported checking their social media accounts approximately every 30 minutes. Conversely, other participants indicated more restricted usage patterns. Participant 6 described a purpose-driven approach to social media use: “I typically use it for studying or researching something specific. I limit my usage to a maximum of one hour,” demonstrating intentional constraints on social media engagement. These narratives illustrate social media usage patterns among middle school students, ranging from frequent, integrated use to more purposeful, limited engagement. The diversity in usage patterns reflects individual differences in approach to and reliance on social media platforms.

#### Content Sharing Patterns

Analysis of students’ social media content-sharing preferences revealed 2 predominant categories: curated content and personal narratives. Participants demonstrated distinct preferences in social media sharing, primarily focusing on personal experiences, recreational interests, and entertainment-oriented content. Specifically, participant 3 expressed a specialized interest in sharing “K-pop edits,” while participant 5 articulated a more personalized approach to platform use, stating, “I share meaningful memories and photographs on Snapchat.” In contrast, several participants reported intentionally limited engagement in content-sharing activities. Participant 7 exemplified this minimal engagement approach: “I do not share content due to restricted phone access.” These divergent sharing patterns illuminate how students express their digital identity and personal interests within social media ecosystems.

#### Perceived Benefits of Social Media Use

Analysis of social media benefits revealed 2 primary thematic categories: educational use and recreational value. Participants consistently identified social media as an instrumental learning resource. Participant 1 emphasized its educational functionality: “It facilitates my learning process and research capabilities.” Additionally, the platforms emerged as significant stress-management mechanisms for students. Participant 4 articulated this therapeutic function, stating, “I utilize it for stress relief during study sessions,” which highlights the role of social media in managing academic stress. Moreover, narratives from participants 3 and 5 indicated that social media makes a substantial contribution to emotional fulfillment and enhances social connections. Corroborating these findings, participants 8 and 9 provided evidence of social media’s positive influence on psychological well-being and its effectiveness as a stress-reduction mechanism. These findings demonstrate the multifaceted role of social media in students’ lives, encompassing educational support, emotional regulation, and social connection. The platforms serve both instrumental purposes in academic contexts and psychological functions in well-being maintenance, suggesting a complex interplay between digital engagement and student development.

#### Family Relationships and Communication Preferences

Examination of family relationships revealed a nuanced landscape of parent-child interactions regarding social media usage. While most participants reported minimal conflict, several participants described significant parental intervention. Participant 5 characterized parental attitudes through explicit concern: “My family strongly disapproves, emphasizing potential harmful effects.” This sentiment was echoed by participant 10, who described persistent parental oversight: “They constantly intervene and criticize.”

Analytical insights from participants 1 and 6 revealed prevalent parental tendencies to restrict and surveil social media use, which frequently led to reduced platform use among students. Participants 8 and 9 conceptualized these parental interventions as constraints on personal autonomy. While parental oversight primarily took restrictive forms, several participants acknowledged that these limitations fostered more conscious platform engagement practices. Communication modality preferences emerged as significant indicators of family dynamics. A distinct bifurcation emerged between participants who favored digital platforms (as exemplified by participants 1 and 4’s preference for WhatsApp) and those who prioritized traditional communication channels (as demonstrated by participants 6 and 7’s preference for face-to-face or telephone communication). These divergent preferences illuminate the complex interplay between established familial communication patterns and evolving individual preferences. These narratives provide contextual support for the quantitative finding that restrictive family relationships were significantly associated with SMUD, suggesting that rigid parental control and limited autonomy may intensify adolescents’ problematic reliance on social media consistent with SMUD patterns.

#### Academic Stress and Social Media Usage

Academic stress emerged as a primary moderating factor in students’ social media engagement patterns. Interview data consistently revealed systematic reductions in social media use during examination periods. Participant 8 articulated this behavioral adaptation: “There is a reduction, and I focus more on my studies,” demonstrating an intentional reduction in digital engagement during academic assessment periods. Participant 10’s narrative highlighted the confluence of multiple pressures: “I am forced to reduce usage due to parental pressure and academic demands,” illustrating how external oversight and academic obligations interact to shape social media consumption patterns. These findings demonstrate the complex interplay between academic pressures and social media engagement, moderated by both internal self-regulatory mechanisms and external parental controls. Participants exhibited adaptive behavioral modifications in their social media engagement patterns in response to academic demands while navigating parental expectations and imposed restrictions.

#### Stress Management Strategies

Analysis of participant responses revealed social media engagement as a predominant coping mechanism for academic stress while simultaneously identifying a diverse array of stress management approaches. The investigation uncovered a comprehensive spectrum of coping strategies, ranging from traditional academic methodologies to contemporary digital engagement practices. Participant narratives demonstrated significant heterogeneity in stress management approaches. Some respondents emphasized conventional stress-reduction techniques, including focused academic preparation and physiological regulation methods, while others primarily used digital platforms to mitigate stress. Participants 1 and 8 exemplified the implementation of multiple concurrent coping mechanisms, incorporating sleep regulation protocols, musical engagement, respiratory exercises, and systematic social media interaction. Participant 10 articulated the psychological displacement function of social media in stress management: “social media provides relief. It helps me forget everything, including thoughts about examinations,” highlighting the platform’s role in facilitating cognitive disengagement from academic stressors. Conversely, a subset of participants, notably participant 6, advocated direct academic engagement as a primary stress-management strategy, asserting, “I achieve success through intensive studying.” This perspective suggests the existence of a student cohort that views academic immersion as the optimal approach to managing academic stress. These divergent approaches illuminate substantial individual variation in stress management preferences and coping mechanisms among students.

The findings demonstrate that while social media is a significant stress-management tool for many students, individual coping strategies remain highly individualized and multidimensional, reflecting the complex nature of academic stress-management approaches in contemporary educational contexts. These qualitative insights also help explain the quantitative association between academic stress and SMUD by showing that students may increasingly use social media as a coping strategy to manage educational pressure. Overall, the qualitative findings functioned as an integrated explanatory layer that triangulated quantitative pathways with adolescents' lived experiences, thereby strengthening mixed methods meta-inferences regarding vulnerability to SMUD.

## Discussion

### Principal Findings

This study examined the associations among SMUD, family relationships, and academic stress in middle school students through the lens of EST. Recent increases in social media engagement and their associated adverse outcomes have become critical considerations in adolescent developmental trajectories [37]. Social media has evolved beyond its primary communication function to become an adaptive coping mechanism and a fundamental component of students’ social ecosystem [38]. By integrating structural pathways with qualitative thematic evidence, the sequential explanatory design enabled formal triangulation of statistical associations with adolescents' lived experiences.

Quantitative analyses yielded support for a positive association between restrictive family relationships and SMUD (H1). This finding aligns closely with Gao et al’s [15] research that established significant correlations between diminished family functioning and problematic social media engagement patterns. Furthermore, a prior study found that adolescents’ social media use was embedded in family and peer connectedness, and that a lack of trust within parent-adolescent relationships could intensify adolescents’ reluctance to disclose online harms, thereby increasing vulnerability in already-strained social environments [39]. These findings suggest that restrictive family relationship dynamics are associated with adolescents’ digital behaviors within the microsystem level of EST.

The nonsignificant effect of supportive family relationships on SMUD (H2) warrants a cautious interpretation. Earlier work has framed family cohesion and parental support as protective against adolescent internet-related problems. Yet, the present results indicate that such relationships may not function as a direct protective route in this sample [22,23]. A contextual reading remains plausible, since supportive family relationships may be fairly common in collectivist settings such as Türkiye [40-42]. However, culture was not measured directly, and this should not be treated as the leading explanation. Measurement factors may also matter, as range restriction on the subscale is possible when the 3-point response format narrows score variability and dulls sensitivity to individual differences. The FRSC was designed for children younger than those in the 11- to 14-year-old age range studied here, leaving its suitability for early adolescents open to further scrutiny. These considerations suggest that the null supportive-family pathway may reflect contextual and measurement conditions rather than the absence of a protective family process.

These findings suggest links among family dynamics, cultural context, and social media engagement patterns. The results demonstrate the complex interactions among system levels within EST in shaping adolescent social media behavior, with particular emphasis on the concurrent influence of microsystem elements (family relationships) and macrosystem factors (cultural context). This theoretical integration offers a comprehensive framework for understanding the complex nature of adolescents’ social media engagement within their broader developmental context.

Qualitative analyses suggested that parental attitudes may shape students’ social media engagement patterns. Multiple participants reported parental restrictions on social media use, indicating a direct impact on engagement duration and patterns. Parental interventions and regulatory mechanisms have influenced students’ social media relationships in a bidirectional manner. While parental risk advisories fostered more judicious usage patterns, stringent restrictions were associated with greater vulnerability to problematic social media use. This finding aligns with the existing literature, which suggests that restrictive family relationships may be associated with increased social media use as an escape mechanism [43].

The demonstrated direct positive relationship between academic stress and SMUD (H3) aligns well with Jun and Choi’s [16] empirical findings. Kardefelt-Winther’s [17] theoretical proposition regarding social media’s functionality as a stress-mitigation mechanism provides substantial support for these observations. Wang et al [25] investigated the relationship between problematic social media engagement and academic stress among Chinese adolescents, revealing a significant association. Correspondingly, Tutgun Ünal [44] identified a significant relationship between social media engagement frequency and heightened academic anxiety and generalized stress [44]. These findings suggested that while social media may function as an immediate stress-amelioration mechanism, it potentially augments long-term addiction susceptibility. Gökbulut and Bilen [45] further articulated the role of social media as an escape mechanism while emphasizing its potential to undermine students’ long-term stress management. Moreover, Chen and Xiao [46] suggest that social media may intensify academic stress through social comparison processes and heightened expectations. These results may be interpreted within EST as suggesting potential interactions between school-related stress and family relational dynamics, although direct mesosystem processes were not empirically measured in this study.

The qualitative findings also refined this interpretation by showing that academic stress was not uniformly linked to greater social media engagement. Some participants described social media as a way to relieve academic pressure, whereas others reported reducing their social media use during examination periods to focus on schoolwork. This tension suggests that academic stress may operate differently depending on its duration and timing. Chronic or generalized academic stress may be associated with greater SMUD symptoms, while acute examination periods may trigger short-term self-regulation among some students. Because the quantitative data were collected at a single time point, the model could not distinguish between chronic academic stress and temporary exam-related regulation. This mixed methods finding therefore complicates the quantitative pathway and points to the need for longitudinal or diary-based studies to examine how academic stress and social media use change throughout the school calendar.

A particularly salient finding reveals the mediating function of restrictive family relationships in the association between academic stress and SMUD (H4). This observation, consistent with Hong et al’s [47] findings regarding the mediating role of family functionality in internet addiction, supports the usefulness of EST as an interpretive framework for understanding these developmental associations. This finding suggests that academic stress was directly associated with SMUD and was also linked to SMUD through restrictive family relationships within the specified model. The longitudinal investigation of Hong Kong adolescents provides additional empirical support for these findings [20].

The nonsignificant mediating role of supportive family relationships in SMUD (H5) should be read alongside this same measurement caution. Prior research suggests that higher-quality parent-child relationships and stronger overall family functioning can lower vulnerability to problematic internet or social media use [21,48]. Yet, the present model did not support a meaningful supportive-family pathway. One reading is that restrictive family relationships act as more proximal and active risk correlates of SMUD than supportive relationships act as direct protective ones. Restrictive dynamics such as excessive control, limited autonomy, and conflict may sit closer to adolescents’ problematic reliance on social media. The null supportive-family pathway may also stem in part from the FRSC’s narrow response range and the still-open question of whether the scale is appropriate for early adolescents. Overall, this pattern suggests that supportive family relationships operate more indirectly or contextually, whereas restrictive family relationships may reflect more immediate routes to vulnerability.

In conclusion, this investigation examined the intricate interrelationships among SMUD, family relationships, and academic stress within the EST theoretical framework. The findings indicate significant associations of restrictive family relationships with SMUD while revealing unexpectedly limited protective effects of supportive family relationships. Furthermore, considering the direct association between academic stress and SMUD, students may increasingly rely on social media engagement as a coping mechanism, potentially heightening SMUD risk. These results highlight the concurrent influence of family dynamics and academic stress on SMUD, supporting the use of EST as a broader interpretive framework for understanding these complex developmental associations. Future cross-cultural investigations are warranted to elucidate the influence of cultural factors and diverse family structures on these relationships.

### Practical Implications

The findings have implications for prevention and intervention strategies addressing SMUD. The observed association between restrictive family relationships and SMUD underscores the importance of family-based prevention and guidance efforts. As suggested by Throuvala et al [3], programs focused on enhancing parenting skills may prove efficacious in SMUD prevention [3]. Moreover, given the significant role of academic stress, educational institutions should integrate comprehensive stress management and digital literacy training into their curricula [14].

The findings suggest the development of targeted strategies for educators and families. Educational institutions should implement comprehensive social media management strategies, including regulatory mechanisms and monitoring protocols, while maintaining appropriate usage boundaries. Educational policy revisions should emphasize the importance of enhancing digital literacy and social media education through comprehensive awareness programs. Furthermore, family practice enhancement should focus on developing supportive guidance strategies and implementing constructive monitoring approaches within balanced regulatory frameworks.

### Limitations and Future Research Directions

The study has several methodological strengths, including the use of an EST-informed framework, a mixed methods design, and the examination of indirect pathways. However, several limitations should be considered. Although EST served as the guiding theoretical framework, direct mesosystem processes, such as family-school interactions, were not empirically measured. Therefore, interpretations of broader system-level processes should be approached with caution. The sample was also drawn from a specific middle school population within a particular cultural and educational context, which may limit the generalizability of the findings to adolescents from different regions, socioeconomic backgrounds, school systems, or cultural settings. In addition, the use of convenience sampling may have introduced selection bias, as participating schools and students may differ from nonparticipating schools and students in terms of school climate, family involvement, academic pressure, or digital media practices.

The cross-sectional design limits causal inference. The hypothesized model was theoretically grounded, but the design could not establish temporal precedence among academic stress, family relationships, and SMUD. The specified ordering should therefore be read as a theoretical modeling assumption rather than as evidence that academic stress precedes restrictive family relationships or that restrictive family relationships precede SMUD. The indirect pathway through restrictive family relationships remains conditional on this assumed sequence. Reverse or reciprocal associations are plausible, including the possibility that higher SMUD elicits greater parental restriction, and future longitudinal designs should test these competing temporal pathways directly.

The study also relied on self-report measures, which may be influenced by social desirability bias, recall bias, and adolescents’ subjective perceptions of their family relationships, academic stress, and social media use. Prior evidence indicates that self-reported digital media use often diverges from logged or actual use, especially when behavioral frequency or problematic use is assessed [49,50]. The observed associations should accordingly be read as perceived and self-reported patterns rather than objective indicators of family processes or actual platform behavior. The SMUD scale, in particular, captured self-reported symptom severity rather than actual exposure, time-stamped use, or behavioral traces. Future studies could pair validated self-report measures with passive sensing, digital trace data, or explainable computational approaches, since earlier work shows that social media language and behavioral histories can support computational detection of mental health-related risk patterns [51,52]. The qualitative phase drew on a single focus group of 10 participants, which narrowed the range of perspectives captured, so these findings are best understood as contextual explanatory insights that refined the quantitative results rather than as broadly transferable qualitative conclusions. The FRSC also uses a 3-point Likert format, which may reduce sensitivity relative to 5- or 7-point scales, and, because it was developed for younger children, its developmental fit for the present sample of 11- to 14-year-olds warrants examination in future validation work.

Future studies should use longitudinal designs to examine the temporal ordering of academic stress, family relationships, and SMUD. Research with larger and more diverse samples would also help clarify the generalizability of these findings across different socioeconomic, cultural, and educational contexts. Future mixed methods studies could include multiple focus groups or individual interviews to examine adolescents’ experiences in greater depth. In addition, combining self-report scales with objective or behavioral indicators, such as digital trace data or platform-use metrics, may provide a more precise understanding of adolescent SMUD and its contextual correlates.

Overall, the findings suggest that academic stress and restrictive family relationships are associated with SMUD among middle school students, while the role of supportive family relationships appears more complex. These results support the value of multidimensional prevention and guidance approaches that consider school pressure, family communication, and adolescents’ digital media practices together.

## Abbreviations

CFA: confirmatory factor analysis
CFI: Comparative Fit Index
EST: ecological systems theory
FRSC: Family Relationships Scale for Children
RMSEA: root-mean-square error of approximation
SEM: structural equation modeling
SMUD: social media use disorder
SRMR: standardized root-mean-square residual
TLI: Tucker-Lewis Index

## Data Availability

The datasets generated or analyzed during this study are available from the corresponding author on reasonable request.
